# Interim analysis for binary outcome trials with a long fixed follow-up time and repeated outcome assessments at pre-specified times

**DOI:** 10.1186/2193-1801-3-323

**Published:** 2014-06-26

**Authors:** Sameer Parpia, Jim A Julian, Chushu Gu, Lehana Thabane, Mark N Levine

**Affiliations:** Ontario Clinical Oncology Group, Department of Oncology, McMaster University, 711 Concession Street – G (60) Wing 1st Floor, Hamilton, ON L8V 1C3 Canada; Biostatistics Unit - FSORC, St Joseph’s Healthcare - Hamilton, 50 Charlton Avenue East, Hamilton, ON L8N 4A6 Canada

**Keywords:** Interim analysis, Binary outcome, Power, Type I error

## Abstract

In trials with binary outcomes, assessed repeatedly at pre-specified times and where the subject is considered to have experienced a failure at the first occurrence of the outcome, interim analyses are performed, generally, after half or more of the subjects have completed follow-up. Depending on the duration of accrual relative to the length of follow-up, this may be inefficient, since there is a possibility that the trial will have completed accrual prior to the interim analysis. An alternative is to plan the interim analysis after subjects have completed follow-up to a time that is less than the fixed full follow-up duration. Using simulations, we evaluated three methods to estimate the event proportion for the interim analysis in terms of type I and II errors and the probability of early stopping. We considered: 1) estimation of the event proportion based on subjects who have been followed for a pre-specified time (less than the full follow-up duration) or who experienced the outcome; 2) estimation of the event proportion based on data from all subjects that have been randomized by the time of the interim analysis; and 3) the Kaplan-Meier approach to estimate the event proportion at the time of the interim analysis. Our results show that all methods preserve and have comparable type I and II errors in certain scenarios. In these cases, we recommend using the Kaplan-Meier method because it incorporates all the available data and has greater probability of early stopping when the treatment effect exists.

## Background

Interim analyses that permit early stopping of a randomized controlled trial (RCT) for extremely positive results or for futility are included in the design for ethical and economic reasons. Strategies have been developed for interim analyses such that the overall type I error of the entire trial is preserved at a fixed level (Haybittle 1971; O'Brien and Fleming 1979; Peto et al. 1976; Pocock 1977).

Often, the primary outcome is whether or not a subject experienced an event over a fixed period of time *T*. In some trials, the outcome is assessed repeatedly at pre-specified times during follow-up, and the subject is considered a failure if the event occurs at any time. For example, in a cardiovascular RCT investigating the effect of an intervention for preventing post-thrombotic syndrome, subjects can be assessed every 6 months for up to 24 months using a disease-specific questionnaire (Enden et al. 2012; Vedantham et al. 2013). A failure has occurred if the questionnaire score exceeds a pre-specified threshold. Another example would be a breast cancer radiotherapy RCT where adverse cosmesis (i.e. a dichotomy), assessed at 1, 3 and 5 years post-randomization, would be the primary safety outcome and the focus of the interim analysis.

Interim analyses are generally performed after half or more of the subjects have completed follow-up (Pedley 2011). Depending on the duration of accrual relative to the length of follow-up, this strategy may be inefficient because it is possible that accrual will have been completed and patients will have finished treatment prior to the interim analysis. If, however, the interim analysis was done earlier and a statistically significant effect was found, the trial may be stopped, and all future subjects would receive the experimental therapy.

In this situation, one alternative is to plan an interim analysis after a smaller percentage of subjects have completed full follow-up. However, there is a low probability of terminating the trial early when the interim analysis is based on so little information, and, therefore, such an analysis would unnecessarily spend alpha (Togo and Iwasaki 2013). A second alternative is to plan the interim analysis after half or more of the subjects have completed a specified portion of the follow-up *R*, where *R* < *T*, and *T* is the fixed full follow-up duration for each subject.

Several researchers have studied methods that combine data from subjects who have completed full follow-up with those who have been followed for duration *R* in situations where the outcome is reversible (Marschner and Becker 2001; Sooriyarachchi et al. 2006; Whitehead et al. 2008). In our research, however, the situation is different in that the outcome can be ascertained at any of the pre-specified visits during follow-up and is irreversible.

In this paper, we consider 3 methods of estimating the interim event proportion (risk) for each treatment group in an RCT for an interim analysis: 1) estimated event proportion based only on subjects who have been followed for at least duration *R* or who had an outcome event; 2) the event proportion based on data from subjects that have been randomized by the time of the interim analysis, and 3) the Kaplan-Meier approach to estimate the event proportion. We investigate the effect of each method on the type I and II errors and the probability of early stopping through computer simulation of various trial scenarios.

## Methods

Consider a trial designed to detect an absolute risk reduction (ARR) between the standard group (*π*_0_) and the experimental group (π_1_) over the time period 0 to *T* using a normal approximation *Z*-test with


where  and  are the observed proportions, *n*_*0*_ and *n*_*1*_ are the group sample sizes, and we are testing the one-sided hypotheses H_0_: *π*_1_ ≥ *π*_0_ versus H_1_: *π*_1_ < *π*_0_. Furthermore, we assume 90% power, an alpha of 0.025 and a 1:1 randomization. Since the normal distribution is symmetric, the p-value for a one-sided test is equivalent to half of the two-sided p-value.

**Figure 1:**
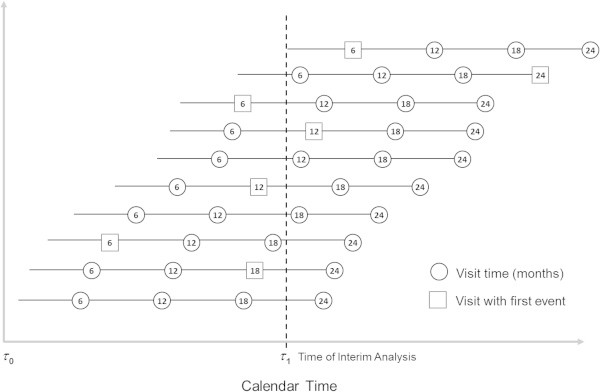
**Plot showing the follow-up time in months for 10 subjects and the proposed time for the interim analysis after 5 (50%) subjects have completed 12 months of follow-up.**

Suppose the trial requires 4 years for enrolment, each subject is followed for 2 years (i.e. *T* = 24 months), and failures are ascertained at any of the four 6-monthly pre-specified visits post-randomization. Let the start of the trial (calendar time) be denoted by *τ*_0_. Following the notation in Table 1, let *t*_*j*_ be the pre-specified visit times in the trial where *t*_*j*_ ≤ *T* and *j* is the visit number where *j* = 0, 1, 2… *J*, and *J* denotes the number of visits (e.g. *J* = 4 and *t*_*0*_ = 0, *t*_*1*_ = 6, *t*_*2*_ = 12, *t*_*3*_ = 18, *t*_*4*_ = 24 months). Suppose an interim analysis is scheduled to occur when 50% of the subjects have completed *R* = 12 months (*t*_*2*_ *= R*) of follow-up which, assuming a uniform recruitment pattern, corresponds to approximately 36 months after the start of the trial, denoted by *τ*_1_ (Figure 1). At the interim analysis, the proportion of subjects who fail in each group could be estimated using any of the following approaches.

**Table 1:** **Notation table for estimation of event proportions**

Visit number ***J***	Visit time ***t***_***j***_	Subjects at risk ***m***_***j***_	New events ***e***_***j***_	Incidence at visit ***j d***_***j***_
0	*t*_*0*_ (<6 m)	*m*_0_	*e*_0_ = 0	*d*_0_ = 0
1	*t*_*1*_ (6 m)	*m*_1_	*e*_1_	*d*_1_ = *e*_1_/*m*_1_
2	*t*_*2*_ (12 m)	*m*_2_	*e*_2_	*d*_2_ = *e*_2_/*m*_2_
3	*t*_*3*_ (18 m)	*m*_3_	*e*_3_	*d*_3_ = *e*_3_/*m*_3_
4	*t*_*4*_ (24 m)	*m*_4_	*e*_4_	*d*_4_ = *e*_4_/*m*_4_

### Method 1: event proportion based on subjects followed for at least duration R or who had an event

In RCTs where the length of enrolment relative to follow-up is not an issue, subjects included in the interim analysis are those who have completed their full follow-up *T* or who have had an event prior to completion (Pedley 2011). A similar approach is used here whereby we include only subjects who have completed at least duration *R* (where *t*_*r*_ = *R, r* refers to the visit at which follow-up time equals *R*) of their full follow-up *T*, or have had an event prior to this point. Since the interim analysis occurs after 50% of the subjects have completed at least follow-up of *R*, this approach includes the first 50% of enrolled subjects plus those subjects that have experienced an event but have not completed follow-up of *R*. For each treatment group *i* (0 = standard, 1 = experimental) at visit time *t*_*j*_, let *m*_*ij*_ be the number of subjects at risk (i.e. have completed visit at *t*_*j*_ without having an event), and let *e*_*ij*_ be the number of new events diagnosed. Then the event proportion in treatment group *i* at the time of interim analysis *τ*_1_ is given by:


The individuals who have experienced an event but have not completed duration *R* of follow-up are included in the numerator and the denominator.

### Method 2: event proportion based on data from subjects that have been randomized by the time of the interim analysis

This simple approach uses data from the subjects randomized by the time of the interim analysis *τ*_1_ (i.e. once 50% of the subjects have been followed for at least time *R*). Let *n*_*i*_ be the number of subjects who have been randomized to treatment group *i*. Then the event proportion for each group at the time of interim analysis *τ*_1_ is given by


which is simply the total number of observed events divided by the number of subjects randomized by time *τ*_1_.

### Method 3: Kaplan-Meier approach

This approach also uses all the data available at the time of the interim analysis *τ*_1_ (i.e. once 50% of the subjects have been followed for at least time *R*). For individuals who have not completed follow-up time *T* (i.e. the full fixed follow-up duration) and have not had the event, they are simply right-censored at the latest time that they were observed. Then the Kaplan-Meier (KM) estimates can be calculated using all randomized subjects and the event proportion in treatment group *i* at the time of interim analysis *τ*_1_ is given by


where *S*_*i*_ (*T*) is the KM survivor function estimate. Following the notation in Table 1, this is equivalent to


We evaluated these methods in terms of overall type I and II errors and the probability of early stopping of the trial for a positive result at the interim. The interim analysis was performed using the Haybittle-Peto (Haybittle 1971; Peto et al. 1976) and O’Brien-Fleming (O'Brien and Fleming 1979) monitoring boundaries for extreme positive results. These boundaries are conservative and require small p-values for early stopping of the trial. Other less conservative boundaries such as the Pocock approach were not evaluated (Freidlin and Korn 2009; Pocock 2005).

### Simulation

We considered six RCTs similar to the trial described in the Methods section (see Table 2). Data for the binary endpoint were generated using the binomial distribution under the null and alternative hypotheses.

**Table 2:** **Summary of six trials considered for simulation with β = 0.10 and a one-sided α = 0.025**

Standard group event proportion (***π***_0_)	Experimental group event proportion (***π***_1_)	Absolute risk reduction (***π***_0_-***π***_1_)	N
0.30	0.25	0.05	3342
0.30	0.20	0.10	796
0.30	0.10	0.20	160
0.50	0.45	0.05	4182
0.50	0.40	0.10	1030
0.50	0.30	0.20	242

For each subject with an event, the time at which the event occurred was randomly assigned to reflect five clinically-plausible scenarios (Table 3), using the following: 1) events were distributed equally across the four time-points with probabilities (0.25, 0.25, 0.25, 0.25) for both groups; 2) the majority of the events occurred in the first two time-points with probabilities (0.35, 0.30, 0.20, 0.15) for both groups; 3) the majority of the events occurred in the last two time-points with probabilities (0.15, 0.20, 0.30, 0.35) for both groups; 4) the standard group follows distribution (3) and the experimental group follows distribution (2); and 5) the reverse of scenario (4). Entry times for subjects over 48 months were randomly generated from a uniform distribution, and the interim analysis was carried out after 50% of the subjects completed *R* = 12 months of follow-up. We carried out 10,000 replications for each trial. Given that *Z* (*x*) and *Z* (*y*) are the interim and final test statistics, respectively, the type I error rate, , and the type II error, , were obtained from data generated under the null and alternative hypotheses, respectively, where *g* and *f* are the interim and final critical values of the O’Brien-Fleming (*g* = 2.797, *f* = 1.977) and Haybittle-Peto (*g* = 3.0, *f* = 1.967) monitoring boundaries. The probability of early stopping, , was obtained under the alternative hypotheses. All analysis was performed in R 2.15 (http://www.r-project.org).

**Table 3:** **Summary of the event distribution probabilities for the simulated scenarios**

Scenario	Event distribution probabilities by visit time	
	***t***_***1***_ ***, t***_***2***_ ***, t***_***3***_ ***, t***_***4***_	
	Standard group	Experimental group
1	0.25, 0.25, 0.25. 0.25	*same as standard*
2	0.35, 0.30, 0.20, 0.15	*same as standard*
3	0.15, 0.20, 0.30, 0.35	*same as standard*
4	0.15, 0.20, 0.30, 0.35	0.35, 0.30, 0.20, 0.15
5	0.35, 0.30, 0.20, 0.15	0.15, 0.20, 0.30, 0.35

## Results

**Figure 2:**
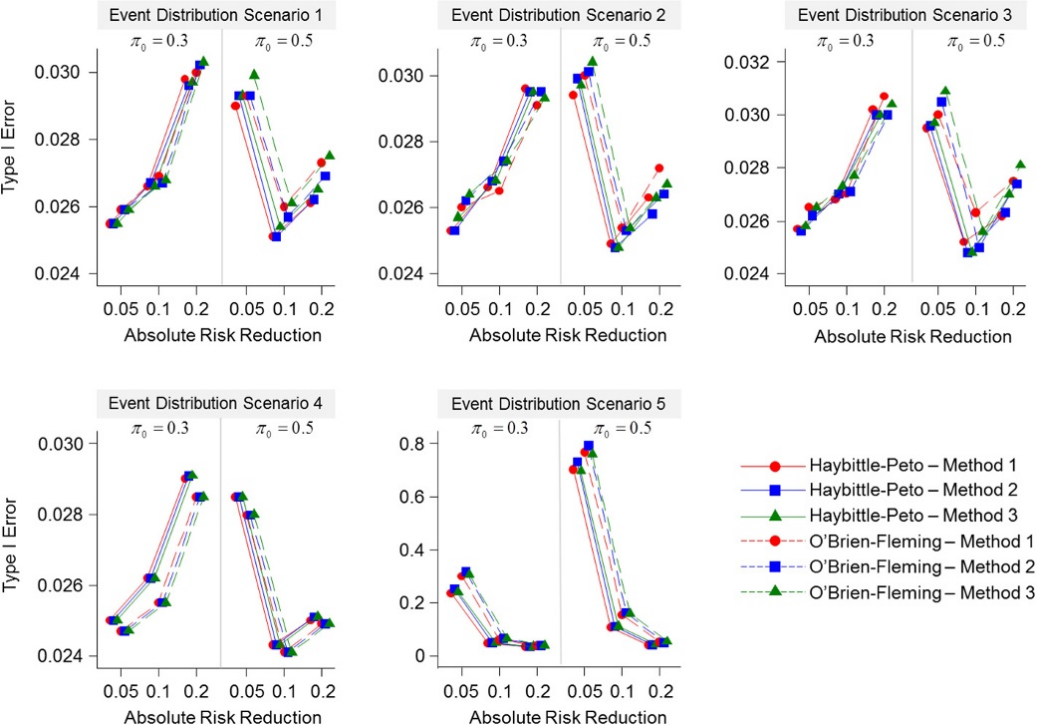
**Overall type I error rates for each trial by event distribution scenario.**

**Figure 3:**
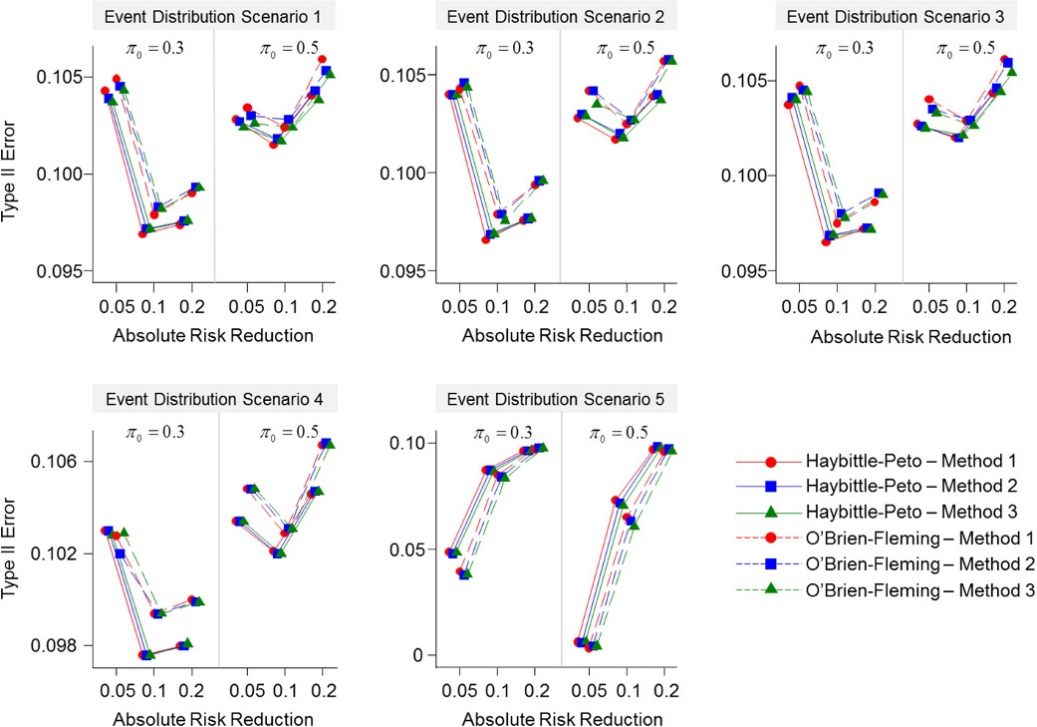
**Overall type II error rates for each trial by event distribution scenario.**

The results of the type I error rates for the three methods are shown graphically in Figure 2. The three methods have comparable type I error rates across each of the trials and event distribution scenarios. The methods in general have nominal or close-to-nominal type I error rates when the event distribution probabilities are equivalent between treatment groups or when the experimental treatment group events occurred earlier in the trial compared with the standard group. However, under these same scenarios, slightly greater-than-nominal type I error rates are seen in the trials where (*π*_*0,*_*π*_*1*_) = (0.30, 0.10) and (*π*_*0,*_*π*_*1*_) = (0.50, 0.45), where the type I error rates are approximately 0.03. For the scenario where the experimental group events occurred later in the trial compared with the standard group, the type I error was generally inflated for all methods.The three methods also have comparable type II error rates (Figure 3). In general, under all event distribution scenarios and trials, the type II error rates are comparable to the nominal value of 0.10 regardless of the interim analysis method or stopping boundary rule. Moreover, in the scenario where the experimental group events occurred later in the trial compared with the standard group, the type II errors rates are much lower than the nominal value for the trials with ARRs of 0.05 and 0.10.

**Figure 4:**
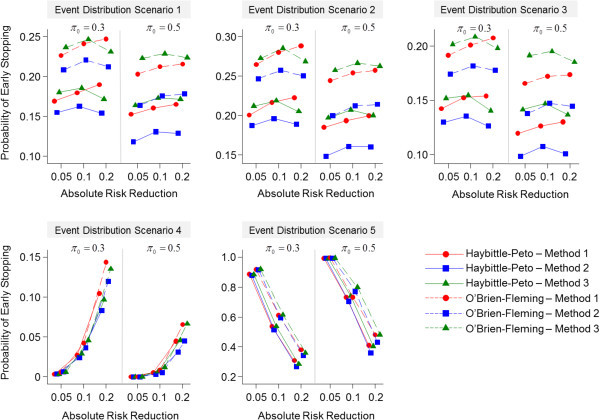
**Probabilities for early stopping under the alternative hypothesis for each trial by event distribution scenario.**

Under the alternative hypothesis, methods 1 and 3 have comparable probabilities for early stopping in scenarios where the treatment groups have equivalent event distributions probabilities over time, specifically in the trials where *π*_*0*_ = 0.30 (Figure 4). Method 3 has a slightly greater probability of early stopping than method 1 in the trials where *π*_*0*_ = 0.50. Moreover, method 2 has the smallest probability of early stopping in scenarios where the treatment groups had equivalent event distributions probabilities over time. On the other hand, all methods have comparable probabilities of early stopping in the scenarios where the treatment groups had contrasting event distributions over time. The highest probabilities for early stopping are seen in the trials where the experimental group had a smaller proportion of events occur earlier in the trial compared with the standard group, and the lowest probabilities of early stopping are seen in the opposite scenario. In general, the probability for early stopping is greater using the O’Brien-Fleming boundaries compared with the Haybittle-Peto monitoring boundaries.

## Discussion

In RCTs with binary endpoints, interim analyses are generally conducted after a considerable percentage of subjects have completed follow-up. However, under certain situations this approach is not optimal since the trial may have completed accrual and all the subjects will have been treated by that time. We evaluated three approaches for an interim analysis when a considerable percentage of subjects complete a follow-up time that is less than the planned trial follow-up.

We observed that the type I error rates were comparable for all three methods. For most trials simulated, under the scenarios where the event distributions were equivalent between treatment groups or the experimental group had events occur earlier than the standard group, the type I error rates were close to the nominal value. These results concur with those of Pedley (2011), who showed that conducting the interim analysis after a considerable percentage of subjects had completed full follow-up (using method 2) produced nominal type 1 error rates, albeit in the situation where events could be measured at any time during follow-up and not just at specific time points. However, we also observed that the type I error rate increased with increasing absolute risk reduction for trials with a standard group event proportion of 0.3, thus resulting in slightly higher type I error rates for the trial with ARR to 0.20. In addition, similar slightly higher type I error rates were seen in the trial with a standard group event proportion of 0.5 and the ARR = 0.05. This is perhaps due to a combination of less variability and a small sample size for the former, and a large sample size and small ARR for the latter. Therefore, trialists should be cautious of using either of these methods under these situations.

While there were situations in which the type I errors were slightly inflated with all methods, the methods performed much better with regard to the type II errors under all scenarios, suggesting that these methods will not have a negative effect on the power to detect the hypothesized difference between treatment groups provided the difference exists. Under the scenarios where the experimental group had events occur later compared with the standard group, the methods showed increased overall power because the probability of early stopping was greater in these scenarios. However, under these scenarios, the type I error rates are inflated.

The methods differed on the probability of early stopping under the alternative hypothesis with method 2 having the lowest probability. This is because this approach includes data from all subjects that have been randomized by the time of the interim analysis in the denominator of the estimation of the event proportion even though a subgroup of these patients would not have had any assessment of the outcome since they would not have reached their first time point for outcome assessment. The consequence is the dilution of the interim treatment effect leading to lower interim power. Method 3 also uses all available data from randomized subjects at the time of the interim analysis. However, it employs a conditional probability approach which differentiates between those subjects who have not yet had an assessment visit (i.e. censored) and who are at risk at each assessment visit, thus yielding a greater probability of early stopping. Similarly, since method 1 uses only a subset of randomized subjects at the time of the interim analysis, the estimated interim treatment effect is less diluted and, therefore, has greater probability for early stopping than method 2. Conversely, since it uses a smaller number of subjects compared with method 3, the probability for early stopping is slightly lower than method 3 in trials where the standard group event proportion is 0.5, because the variability is greater for proportions closer to 0.5. Furthermore, we observed that the probabilities for early stopping are greater using the O’Brien-Fleming boundary compared with the Haybittle-Peto boundary since it is less conservative.

Although the largest probabilities of early stopping under the alternative hypothesis and the smallest type II errors were seen under the scenario where the experimental group had events occurring later compared with the standard group, the type I errors is greatly inflated and, therefore, none of the methods can be recommended in this situation. Since there is a delay in occurrence of the event in the experimental group, this may be perceived as an effect of treatment. However, in situations where investigators are interested in the occurrence of an event over a fixed time period, this scenario, although rare, would still be considered under the null hypothesis.

Our study had some limitations. The generalizability of our findings may be limited since we evaluated six trial scenarios with particular event distributions over time. In diseases where the event distributions over time differ from the ones evaluated in this research, further simulations would be required to evaluate these methods. Secondly, we evaluated trials with one interim analysis after 50% of the subjects completed 12 months of follow-up using the O’Brien-Fleming or Haybittle-Peto approach. These findings may not be applicable to trials in which interim analyses are required at multiple times or when using the alpha spending function approach to monitor the trial. Finally, the biases of the interim event proportions and treatment effects were not evaluated primarily because it is well known that estimators at the interim are biased, especially for estimators that allow for early stopping for positive results. However, further investigation on the estimators is needed.

## Conclusion

Nonetheless, we have shown that under certain scenarios, conducting an interim analysis when a considerable number of subjects have some follow-up data, using any of the methods, preserves the type I and II errors. Although all three methods preserve type I and II errors under these scenarios, we recommend using the Kaplan-Meier method because it incorporates all the available data and has greater probability of early stopping when the treatment effect exists. We have also shown that under certain scenarios, none of these methods is suitable for an interim analysis, and trialists should be cautious when using them. Finally, when possible, an interim analysis should be undertaken when data from a considerable number of subjects who have completed full follow-up are available. However, if waiting for a considerable number of subjects to complete full follow-up is not an efficient approach, such as in the examples described, the methods outlines in this paper should be considered and evaluated to fit the specific needs of the trial.
